# Impacts of COVID-19 on career choices in health professionals and medical students

**DOI:** 10.1186/s12909-023-04328-8

**Published:** 2023-05-26

**Authors:** Linh Phuong Doan, Vu Anh Trong Dam, Laurent Boyer, Pascal Auquier, Guillaume Fond, Bach Tran, Thuc Minh Thi Vu, Hoa Thi Do, Carl A. Latkin, Melvyn W.B. Zhang, Roger C.M. Ho, Cyrus S.H. Ho

**Affiliations:** 1 Institute for Global Health Innovations, Duy Tan University, Da Nang, 550000 Vietnam; 2 Faculty of Medicine, Duy Tan University, Da Nang, 550000 Vietnam; 3 Research Centre on Health Services and Quality of Life, Aix Marseille University, 27, boulevard Jean-Moulin, Marseille cedex 05, 13385 France; 4 Institute of Health Economics and Technology, Hanoi, 100000 Vietnam; 5 Bloomberg School of Public Health, Johns Hopkins University, Baltimore, MD 21205 USA; 6 Lee Kong Chian School of Medicine, Nanyang Technological University Singapore, Singapore, Singapore; 7 Department of Psychological Medicine, Yong Loo Lin School of Medicine, National University of Singapore, Singapore, 119228 Singapore; 8 Institute for Health Innovation and Technology (iHealthtech), National University of Singapore, Singapore, 119077 Singapore

**Keywords:** Career choice, Medical student, Healthcare worker, COVID-19, Vietnam

## Abstract

**Backgrounds:**

The COVID-19 pandemic has resulted in not only significant mortalities in Vietnam but has had an impact on its economy. Previous studies have highlighted how the pandemic has had a marginal impact on Vietnamese healthcare workers working at the frontlines. To date, there have been several other studies examining the impact of COVID-19 on intentions to transition between jobs among healthcare professionals, but this has yet to be explored amongst Vietnamese healthcare workers.

**Methods:**

To achieve the study’s objectives an online cross-sectional study was conducted between September to November 2021. Snowball sampling methodology was adopted for the recruitment of participants. The questionnaire that was used for this study comprised of the following sections: (a) socio-demographic information; (b) impact of COVID-19 on work; (c) risk of exposure to COVID-19; (d) career choices/intentions to change job, and (e) motivation at work.

**Results:**

There were 5727 completed the entire survey. 17.2% of the respondents have had increased job satisfaction, 26.4% reported increased motivation to work, and 40.9% reported decreased motivation to work. Whilst there were changes in the daily work intensity and the level of work-related stress, more than 60% of respondents we sampled did not intend to switch careers. Demographic variables like gender, whether one was a student or an existing healthcare worker, and income related to work motivation. The community’s stigma was a negative factor that declined intrinsic motivation as well as decreased work retention.

**Conclusions:**

Our study is instrumental in identifying the impact of COVID-19 on career choices amongst Vietnamese healthcare workers. The factors identified have clear implications for policymaking.

**Supplementary Information:**

The online version contains supplementary material available at 10.1186/s12909-023-04328-8.

## Introduction

It has been nearly two years since the detection of the first case of COVID-19 in Vietnam [1]. The very first case of COVID-19 was detected on the 23rd of January 2020. Since the onset of the COVID-19 pandemic, Vietnam has seen and undergone a total of 4 waves of infection/reinfection of the COVID-19 virus and its variants [1]. There has been significant mortality since the onset of the pandemic, with approximately 43,063 deaths in Vietnam to date, with a total infection of 10,690.471 cases. The total global number of mortalities due to COVID-19 has to date (as of 20th May 2022) amounted to 626,170 deaths [2]. To an extent, the efforts undertaken by the Vietnamese government have paid off, as it helped in the rapid control of the spread of the virus in each of the waves of reinfection. Measures undertaken by the government ranged from that social distancing to those total lockdowns at the peak of the infections [1]. Apart from the direct impact of the pandemic on the life of individuals, the onset of the pandemic has had a high impact on Vietnam’s economy. Huong et al. (2020) in their article that examined the economic and employment issues in Vietnam, 6 months after the first infection, reported that the onset of the pandemic has resulted in an increase in the rates of unemployment in Vietnam, and in fact, the rates were the highest in the last ten years [3]. Currently, in 2022, as the global pandemic situation is stabilizing, the Vietnamese economy is still recovering, and the projected growth rate for its gross domestic product remains to be lower than what it was before the pandemic [4].

When considering the impact of the pandemic on the population, those working at the frontlines, those in healthcare, and even those who are preparing to make a transition into healthcare are most affected. In a scoping review that was published in 2020 that examined the physical and mental health aspects of COVID-19 on healthcare workers, the authors reported that those working at the frontline are at heightened risk of experiencing both physical and mental health consequences, given that they are directly providing care [5]. Factors leading healthcare workers to suffer from a COVID-19 health impact include having to work in a high-risk environment, having had a family member diagnosed with COVID-19, having suboptimal hand hygiene, improper use of personal protective equipment, close contact with patients, and long daily contact hours [5]. The authors also reported that healthcare workers suffered not only from high levels of depression but were anxious and experienced insomnia and distress [5]. With regard to healthcare workers in Vietnam, Pham et al. (2021) have previously examined the impact of COVID-19 on healthcare workers during the nationwide partial lockdown phase [6]. The authors reported there being the only marginal impact of COVID-19 on the work and life of healthcare workers in Vietnam, though it was highlighted that only 3.2% of the respondents did feel that their work was recognized by Vietnamese society.

Whilst there has been the examination of the impact of COVID-19 on the physical, mental health well-being of healthcare professionals, and studies examining the changes to work during the pandemic in Vietnam, there remains no study that has assessed the impact of COVID-19 on career choices and intentions to undertake career transitions amongst Vietnamese healthcare professionals. In contrast, in the existing literature, there have been several studies that have examined the impact of COVID-19 on career choices, but mainly these studies were performed on medical students or healthcare workers separately. Wang XL et al. (2022) examined the career intentions amongst a group of medical students from Hubei Providence in China, and they reported that several factors mediated students’ decisions about their career choices. These factors include their year/grade, their attitude toward healthcare, and how the pandemic has affected their lives [7]. Deng J et al. [8], in their study which sampled a total of 1837 medical students, reported that 6.9% of those who were currently training to be a medical doctor, have had decreased willingness to be a doctor since the onset of the pandemic. They reported several variables that contributed to one’s willingness to continue their training as a doctor, and these include that of younger age, lower household income, fewer depressive symptoms, those were less exposed to negative pandemic information, and those who were more satisfied with their own major after the pandemic [8]. Bai et al. (2021) in their article explored the career choices of nursing students during the COVID-19 pandemic in China [9]. Of significance, the authors reported that there was an increase in individuals choosing nursing as a career, from 50.9% before the pandemic to 62.7% post-pandemic. One of the reasons that might account for this was how the images of nursing changed when the public media opened and applauded those who served at the frontline, and most notably, policies were being implemented that helped to improve the welfare of nurses [9].

Given this, it is evident that there remain to be several research gaps. While there has been an exploration of the impact of COVID-19 on the healthcare workers in Vietnam, that previous study was limited as it assessed the impacts during the period of the partial lockdown. Also, the previous study has not examined if there was an impact of COVID-19 on career choices. It is important to understand the core mechanism underlying the impact of COVID-19 on work motivation. Our study was developed on the Self-Determination Theory (SDT)– by Edward Deci & Richard Ryan. SDT comprises two vital factors - intrinsic motivation and extrinsic motivation [10]. Intrinsic motivation is the outcome of the feeling of competence (being contented with skills and behaviors), autonomy (having control over our lives), and relatedness (being associated with others) [11]. In contrast, extrinsic motivation is affected by the possibility of receiving rewards, which can be either extrinsic (such as a salary increase) or intrinsic (such as recognition for doing a job well) [11]. With the shortage of human resources in Vietnam, aggravated by the impact of COVID-19, the study was conducted to provide evidence on the change in the nature of work, especially work motivation and the career choices of healthcare workers in Vietnam during COVID-19, not only for healthcare workers but also for the future medical human resources, that is medical students.

## Methods

### Study design and sampling methods

To achieve the study’s objectives an online cross-sectional study was conducted between September to November 2021. This coincided with the 4^th^ wave of the COVID-19 pandemic that was affecting Vietnam. Snowball sampling methodology was adopted for the recruitment of participants.

A questionnaire was conceptualized and was initially tested amongst 15 medical students and staff members of the Vietnam Young Physicians Association. They provided inputs in refining the questionnaire. A questionnaire link was generated, and this link was disseminated to a group of 20 individuals (which comprised medical students and healthcare workers). The questionnaire was stored and disseminated using Survey Monkey’s web platform. Respondents who completed the survey were asked to invite more acquaintances and colleagues. Participants took approximately 30 min and then complete the questionnaire. Before the commencement of the study, all participants were informed of the benefits and risks of participation; and that they could withdraw from the study at any time if they wish to.

### Participants

Criteria for selecting the subjects were as follows: Participants were recruited if they met the following inclusion criteria: (a) aged 16 years and above; (b) currently working at medical universities or other facilities across the country; (c) able to provide informed consent; (d) have access to a computer/smartphone that allows for the completion of the questionnaire.

### Measurements

Our process for creating research tools followed a structured approach. Initially, we conducted a literature review to identify crucial areas of focus and gaps in existing research. Following this, we designed a comprehensive instrument that addressed all relevant topics based on the Vietnam context. Finally, we sought the input of COVID-19 specialists in public health, infectious diseases, health services, and policymaking. These experts, who represent our targeted audience, collaborated with us during the translation, refinement, testing and simplification of the questionnaire. Finally, we utilized the questionnaire that comprised the following sections: (a) socio-demographic information [7, 12]; (b) impact of COVID-19 on work [12, 13]; (c) risk of exposure to COVID-19 [7]; (d) career choices/intentions to change job and (e) motivation at work [14].

***The section on (a)*** socio-demographic information comprised the following questions: age, gender (male/female), marital status (single, others), living location (urban areas, town, rural/mountainous areas), current major (students, nurse/technician, doctor, and others), and type of health facilities (national hospital, provincial/city hospital, university hospital, others).

***The section on (b)*** Impact of COVID-19 on work asked questions relating to current work conditions and how COVID-19 has impacted work. Questions were asked about existing working experience (students, less than one year, from one to five years, more than five years), whether one was still a student or a senior member at his/her workplace (students, below five years, above five years), part-time job (yes/no), number of on-call shifts per week (None, one day, two days, more than two days), average working time per day (less than eight hours, eight hours, and more than eight hours), changes to the average workload (no change, increased < 20%, increased from 20 to 50%, and more than 50%), and changes to the average workload per day (no change, increased < 20%, increased from 20 to 50%, and more than 50%). Further questions were asked to determine if there were any changes in the intensity of work and job requirements. These questions included:

1. Daily work intensity.
2. Level of work-related stress and fatigue.
3. Health risks caused by work.
4. The community’s stigma with the work I’m doing.
5. Your ability to endure and cope with external work pressures.
6. Process and professionalism of routine work.
7. Complexity in coordination between colleagues, and between departments.
8. New knowledge and skills for work.
9. Ability to complete assigned tasks.
10. Ability to ensure safe means of work.

Each of the questions was rated on a five-point Likert Scale (1 = Completely unchanged; 2 = Changed little; 3 = Changed relatively much; 4 = Changed a lot; 5 = Changed extremely, beyond processing capacity).

***The section on (c)*** risk of exposure to COVID-19: COVID-19 prevention includes all pharmaceutical and non-pharmaceutical interventions, such as social distancing, lockdown, wearing masks and other hygiene measures, communication campaigns, vaccination, and care. The section on risk of exposure to COVID-19 included questions that asked the following: participating in COVID-19 pandemic prevention (None, less than one month, from one to three months, more than three months), whether one was vaccinated against COVID-19 (none, one dose, two doses), their current risk of exposure to COVID-19 (none, daily, several times/week, seldom, do not know), self-assessment of their risk of COVID-19 (none, low risk, moderate risk, high risk, infected with COVID-19 / Maybe infected), whether there were any changes in their job satisfaction (decreased, unchanged, increased), and changes in work motivation (decreased, unchanged, increased.

***The section on (d)*** work motivation, we adopted and use the Work Motivation Scale. This measured health professionals’ and medical students’ motivation [14]. There were15 questions that correspond to three domains including intrinsic motivation (7 items), self-worth motivation (4 items), and economic motivation (four items). Each item was scored from 0 “Totally unimportant” to 10 “Extremely important”. Hence, the total score of the three domains was 70, 40, and 40, respectively. The Cronbach’s alpha of the three domains was 0.93, 0.86, and 0.86. The questionnaire included these 15 questions (Appendix 1).

We utilized a 5-Likert question from 1 “Certainly not” to 5 “Definitely yes” to ascertain the participants’ occupational intention as follows: “Continue to stick with the job at the current unit and be determined to complete the task with the highest ability”. Participants who had a higher score indicated higher work retention.

#### Outcome variables and co-variates

The main outcome variables we looked at were the motivation for work and occupational intentions. The covariates that we considered included socio-economic status, working characteristics, and the impact of COVID-19 on work.

### Data analysis

Data analyses were performed using STATA version 16 (Stata Corp. LP, College Station, United States of America). With regard to the handling of missing data, we used the Listwise Deletion method to clean data before analyzing it. Continuous variables were presented as mean and standard deviation (SD), while categorical variables were presented as frequencies with percentages. We used the Chi-squared and Wicolxon-Man-Whitney Tests to test the difference between participants who committed to their current job and were not or unsure.

In this study, we conducted a factor analysis to provide a more comprehensive understanding of the underlying constructs of work motivation of healthcare workers and medical students. There are four main benefits of conducting factor analysis included: (1) To confirm the validity of a scale or questionnaire, (2)To reduce the number of variables, (3) To identify interrelationships among variables, and (4) To develop hypotheses for future research.

#### Reliability

The internal consistency reliability was checked by calculating Cronbach’s alpha. The alpha value of 0.7 or above was considered an acceptable [15]. In terms of work motivation, Cronbach’s alpha of the three sub-scales was good at 0.93, 0.86, and 0.89, respectively.

#### Factorial structure

The Exploratory Factor Analysis (EFA) using principal component analysis (PCA) was performed to evaluate the optimal structural model of the instrument according to the observed data. The number of factors was determined based on the Scree plot, and parallel analysis, along with eigenvalues and the proportion of variance explained [16]. Items with a loading value ≥ of 0.4 were included in the relevant components [16]. After applying EFA, the optimal structural model of work motivation has three domains including intrinsic motivation (seven items), self-worth motivation (four items), and economic motivation (four items).

Potential covariates for full models’ motivation of work and commitment to current job included individual characteristics, working characteristics, and impact of COVID-19 on work. Multivariate Tobit regression was used to determine factors related to the score of three domains of motivation of work. We used multivariate ordinal logit regression to confirm factors associated with commitment to the current job. These models were then combined with the stepwise forward strategies to produce reduced models with p < 0.2 as the threshold for included variables. The p-value (P) < 0.05 was considered statistically significant.

## Results

There were 5839 participants who returned the questionnaire, but only 5727 completed the entire survey. The completion rate was 98.1%.

Table 1 presents the results obtained from the analysis of demographic characteristics. The majority of participants commit to their current job (63.8%). Most participants were between 21 and 25 years old (41.5%). There were 69.2% female participants, 85.7% participants were singled/divorced/widowed, and most (78.8%) of those were students. Most of the participants had a mean income per month of under 5 million VND (82.1%) and their monthly household income per capita was also under 5 million VND (48.6%). There were 54.4% of the participants worked in a university hospital and 33.9% of those worked in provincial/city hospitals. 13.5% of participants were working on a part-time basis. Participants who were 16–20 years old, were female, married, had monthly income and monthly household income per month higher than 5 million VND, worked in the national hospital had higher prevalence commitment to their current job, the statistically significant difference with p < 0.05.

**Table 1:** Individual characteristics of participants

Characteristics	Not commit to current job/Unsure		Commit to current job		Total		p-value
	n	%	n	%	n	%	
**Total**	2071	36.2	3656	63.8	5727	100.0	
**Age group**							
16–20 years old	809	39.2	1491	40.9	2300	40.3	< 0.001
21–25 years old	955	46.3	1411	38.7	2366	41.5	
More than years old	299	14.5	741	20.3	1040	18.2	
**Gender**							
Male	676	32.9	1075	29.6	1751	30.8	0.01
Female	1381	67.1	2556	70.4	3937	69.2	
**Marital status**							
Singled/Divorced/Widowed	1833	89.4	3024	83.6	4857	85.7	< 0.001
Married	218	10.6	595	16.4	813	14.3	
**Living location**							
Rural areas	1408	68.1	2229	61.1	3637	63.7	< 0.001
Town	254	12.3	519	14.2	773	13.5	
Urban/Mountainous/Island	406	19.6	898	24.6	1304	22.8	
**Occupation**							
Student	1688	81.7	2810	77.1	4498	78.8	< 0.001
Nurse, technician	147	7.1	344	9.4	491	8.6	
Doctor	194	9.4	380	10.4	574	10.1	
Other	36	1.7	110	3.0	146	2.6	
**Mean income/month**							
Under 5 million VND	1693	84.8	2865	80.6	4558	82.1	< 0.001
5–10 million VND	221	11.1	522	14.7	743	13.4	
10 million VND or above	82	4.1	167	4.7	249	4.5	
**Monthly household income per capita**							
Under 5 million VND	1076	52.7	1674	46.3	2750	48.6	< 0.001
5–10 million VND	601	29.5	1255	34.7	1856	32.8	
10 million VND or above	363	17.8	685	19.0	1048	18.5	
**Type of workplace**							
Student	1398	67.7	2321	63.7	3719	65.2	< 0.001
University hospital	354	17.2	608	16.7	962	16.9	
General hospital	175	8.5	446	12.2	621	10.9	
CDC/Medical center/Public health station	86	4.2	178	4.9	264	4.6	
Other	51	2.5	88	2.4	139	2.4	
**Levels of health facilities**							
National Hospital	155	7.5	346	9.5	501	8.8	0.04
Provincial/city hospital	715	34.8	1216	33.5	1931	33.9	
University hospital	1116	54.3	1977	54.4	3093	54.4	
Other	68	3.3	96	2.6	164	2.9	
**Working experience**							
Students	1424	69.1	2379	65.3	3803	66.6	< 0.001
< 1 years	108	5.2	136	3.7	244	4.3	
1–5 years	320	15.5	542	14.9	862	15.1	
More than 5 years	210	10.2	587	16.1	797	14.0	
**Having part-time job**	298	14.5	468	13.0	766	13.5	0.10
**Work retention**							
**Total**	2071	36.2	3656	63.8	5727	100.0	
**Age group**							
16–20 years old	809	39.2	1491	40.9	2300	40.3	< 0.001
21–25 years old	955	46.3	1411	38.7	2366	41.5	
More than 25 years old	299	14.5	741	20.3	1040	18.2	
**Gender**							
Male	676	32.9	1075	29.6	1751	30.8	0.01
Female	1381	67.1	2556	70.4	3937	69.2	
**Marital status**							
Singled/Divorced/Widowed	1833	89.4	3024	83.6	4857	85.7	< 0.001
Married	218	10.6	595	16.4	813	14.3	
**Living location**							
Rural areas	1408	68.1	2229	61.1	3637	63.7	< 0.001
Town	254	12.3	519	14.2	773	13.5	
Urban/Mountainous/Island	406	19.6	898	24.6	1304	22.8	
**Occupation**							
Student	1688	81.7	2810	77.1	4498	78.8	< 0.001
Nurse, technician	147	7.1	344	9.4	491	8.6	
Doctor	194	9.4	380	10.4	574	10.1	
Other	36	1.7	110	3.0	146	2.6	
**Mean income/month**							
Under 5 million VND	1693	84.8	2865	80.6	4558	82.1	< 0.001
5–10 million VND	221	11.1	522	14.7	743	13.4	
10 million VND or above	82	4.1	167	4.7	249	4.5	
**Monthly household income per capita**							
Under 5 million VND	1076	52.7	1674	46.3	2750	48.6	< 0.001
5–10 million VND	601	29.5	1255	34.7	1856	32.8	
10 million VND or above	363	17.8	685	19.0	1048	18.5	
**Type of workplace**							
Student	1398	67.7	2321	63.7	3719	65.2	< 0.001
University hospital	354	17.2	608	16.7	962	16.9	
General hospital	175	8.5	446	12.2	621	10.9	
CDC/Medical center/Public health station	86	4.2	178	4.9	264	4.6	
Other	51	2.5	88	2.4	139	2.4	
**Levels of health facilities**							
National Hospital	155	7.5	346	9.5	501	8.8	0.04
Provincial/city hospital	715	34.8	1216	33.5	1931	33.9	
University hospital	1116	54.3	1977	54.4	3093	54.4	
Other	68	3.3	96	2.6	164	2.9	
**Working experience**							
Students	1424	69.1	2379	65.3	3803	66.6	< 0.001
< 1 years	108	5.2	136	3.7	244	4.3	
1–5 years	320	15.5	542	14.9	862	15.1	
More than 5 years	210	10.2	587	16.1	797	14.0	
**Having part-time job**	298	14.5	468	13.0	766	13.5	0.10

Table 2 provides a summary of the impact of COVID-19 on work and one’s risk of exposure to COVID-19. There were 47.7% of participants do not participate in COVID-19 pandemic prevention, and 27% of those participate in medical examination or treatment. Approximately two-thirds of the participants reported that they did not have to fulfill any on-call shifts. More than 50% of participants work less than 8 h per day (56.2%), and around one-third of respondents reported an increase in average working time and workload. Only 17.2% of participants had increased job satisfaction, and 26.4% of participants had increased job motivation. Half of the participants got 2 doses of the COVID-19 vaccine (50.3%), and 41.5% of participants assessed they had a low risk of COVID-19.

**Table 2:** Impact of COVID-19 on work and risk of exposure to COVID-19 of participants

Characteristics	Not commit to current job/Unsure		Commit to current job		Total		p-value
	n	%	n	%	n	%	
**Participate in COVID-19 pandemic prevention**							
Do not participate in	959	46.6	1762	48.4	2721	47.7	0.004
< 1 month	349	16.9	552	15.1	901	15.8	
1–3 months	549	26.7	884	24.3	1433	25.1	
> 3 months	202	9.8	446	12.2	648	11.4	
**Participating in medical examination or treatment**	543	26.4	996	27.4	1539	27.0	0.41
**Number of on-call shifts per week**							
None	1415	68.8	2435	67.1	3850	67.7	0.58
1 day	272	13.2	498	13.7	770	13.5	
2 days	176	8.6	317	8.7	493	8.7	
> 2 days	195	9.5	378	10.4	573	10.1	
**Average working time per day**							
< 8 h	1219	59.7	1943	54.2	3162	56.2	< 0.001
8 h	494	24.2	983	27.4	1477	26.3	
> 8 h	328	16.1	659	18.4	987	17.5	
**Having part-time job**	298	14.5	468	13.0	766	13.5	0.10
**Average workload increased**							
No changed	1420	69.7	2398	66.8	3818	67.9	0.12
Increased < 20%	309	15.2	571	15.9	880	15.6	
Increased 20-<50%	193	9.5	397	11.1	590	10.5	
More than 50%	115	5.6	223	6.2	338	6.0	
**Average time to work increased**							
No changed	1422	69.7	2405	67.0	3827	68.0	0.14
Increased < 20%	329	16.1	619	17.2	948	16.8	
Increased 20-<50%	182	8.9	376	10.5	558	9.9	
More than 50%	106	5.2	191	5.3	297	5.3	
**Change in job satisfaction**							
Decreased	899	43.8	1796	49.4	2695	47.4	< 0.001
Unchanged	901	43.9	1112	30.6	2013	35.4	
Increased	254	12.4	728	20.0	982	17.3	
**Change in work motivation**							
Decreased	828	40.1	1503	41.3	2331	40.9	< 0.001
Unchanged	872	42.3	992	27.3	1864	32.7	
Increased	363	17.6	1145	31.5	1508	26.4	
**Vaccinated against COVID-19**							
None	123	9.6	204	9.2	327	9.3	0.84
1 dose	519	40.6	897	40.2	1416	40.4	
2 doses	635	49.7	1128	50.6	1763	50.3	
**The current risk of exposure to COVID-19**							
None	560	43.8	912	40.8	1472	41.9	0.003
Daily	159	12.4	378	16.9	537	15.3	
Several times/weeks	84	6.6	128	5.7	212	6.0	
Seldom	216	16.9	409	18.3	625	17.8	
Do not know	260	20.3	407	18.2	667	19.0	
**Self-assess risk of COVID-19**							
None	338	26.5	574	25.7	912	26.0	0.002
Low risk	521	40.8	934	41.8	1455	41.5	
Moderate risk	257	20.1	392	17.6	649	18.5	
High risk	111	8.7	274	12.3	385	11.0	
Infected with COVID19/Maybe infected	50	3.9	59	2.6	109	3.1	
**Work retention**							
**Participate in COVID-19 pandemic prevention**							
Do not participate in	959	46.6	1762	48.4	2721	47.7	0.004
< 1 month	349	16.9	552	15.1	901	15.8	
1–3 months	549	26.7	884	24.3	1433	25.1	
> 3 months	202	9.8	446	12.2	648	11.4	
**Participating in medical examination or treatment**	543	26.4	996	27.4	1539	27.0	0.41
**Number of on-call shifts per week**							
None	1415	68.8	2435	67.1	3850	67.7	0.58
1 day	272	13.2	498	13.7	770	13.5	
2 days	176	8.6	317	8.7	493	8.7	
> 2 days	195	9.5	378	10.4	573	10.1	
**Average working time per day**							
< 8 h	1219	59.7	1943	54.2	3162	56.2	< 0.001
8 h	494	24.2	983	27.4	1477	26.3	
> 8 h	328	16.1	659	18.4	987	17.5	
**Average workload increased**							
No changed	1420	69.7	2398	66.8	3818	67.9	0.12
Increased < 20%	309	15.2	571	15.9	880	15.6	
Increased 20-<50%	193	9.5 h	397	11.1	590	10.5	
More than 50%	115	5.6	223	6.2	338	6.0	
**Average time to work increased**							
No changed	1422	69.7	2405	67.0	3827	68.0	0.14
Increased < 20%	329	16.1	619	17.2	948	16.8	
Increased 20-<50%	182	8.9	376	10.5	558	9.9	
More than 50%	106	5.2	191	5.3	297	5.3	
**Change in job satisfaction**							
Decreased	899	43.8	1796	49.4	2695	47.4	< 0.001
Unchanged	901	43.9	1112	30.6	2013	35.4	
Increased	254	12.4	728	20.0	982	17.2	
**Change in work motivation**							
Decreased	828	40.1	1503	41.3	2331	40.9	< 0.001
Unchanged	872	42.3	992	27.3	1864	32.7	
Increased	363	d	1145	31.5	1508	26.4	
**Vaccinated against COVID-19**							
None	123	9.6	204	9.2	327	9.3	0.84
1 dose	519	40.6	897	40.2	1416	40.4	
2 doses	635	49.7	1128	50.6	1763	50.3	
**The current risk of exposure to COVID-19**							
None	560	43.8	912	40.8	1472	41.9	0.003
Daily	159	12.4	378	16.9	537	15.3	
Several times/weeks	84	6.6	128	5.7	212	6.0	
Seldom	216	16.9	409	18.3	625	17.8	
Do not know	260	20.3	407	18.2	667	19.0	
**Self-assess risk of COVID-19**							
None	338	26.5	574	25.7	912	26.0	0.002
Low risk	521	40.8	934	41.8	1455	41.5	
Moderate risk	257	20.1	392	17.6	649	18.5	
High risk	111	8.7	274	12.3	385	11.0	
Infected with COVID19/Maybe infected	50	3.9	59	2.6	109	3.1	

Table 3 provided the construct reliability and validity of factors from the Work Motivation Scale that health professionals and medical students completed. 3 dimensions were classified namely “Intrinsic motivation”, “Self-worth motivation”, and economic motivation”. Cronbach’s alpha ranges from 0.86 to 0.93 and it stabilized across domains.

**Table 3:** Factor loadings of work motivation of participants

Items	Mean (SD)	Intrinsic motivation	Self-worth motivation	Economic motivation
1. Because I enjoy doing what I do at work every day.	6.6 (2.6)	0.77		
2. Because I enjoy my work tasks.	6.9 (2.4)	0.88		
3. Because the work that I do is very interesting.	6.6 (2.4)	0.83		
4. Because being a health worker is a fundamental part of who I am	7.3 (2.5)	0.76		
5. Because my work is extremely important for my patients	7.4 (2.5)	0.67		
6. Because I want to make a difference in people’s live	6.7 (2.6)	0.59		
7. To feel good about myself	7.3 (2.4)	0.59		
8. Because my reputation depends on my work.	5.7 (2.9)		0.62	
9. Because of the appreciation I receive from my patients and the community	6.2 (2.7)		0.71	
10. Do not let my team down.	7.1 (2.4)		0.54	
11. Because my supervisor recognizes and appreciates me	6.3 (2.6)		0.68	
12. Because of the benefits that come with my job	6.2 (2.7)			0.54
13. To be able to provide for my family	6.4 (3.1)			0.83
14. Because of the financial security my job provides me with	6.2 (3.0)			0.83
15. To earn money	6.1 (3.1)			0.80
**Reliability**				
Cronbach’s alpha		0.93	0.86	0.89
**Domains scores**				
**Mean**		48.7	25.1	24.7
**SD**		14.6	9.1	10.4

Table 4 presented the recent changes in work and work motivation regarding work retention characteristics. In terms of work retention, more than 60% of participants commit to their current job (36.04% very much possibility and 27.8% definitely yes). Regarding recent changes in work, new knowledge and skills for work had the highest score (2.55 ± 1.02), followed by daily work intensity (2.53 ± 1.00) and Level of work-related stress and fatigue (2.52 ± 1.02). The mean score of intrínic, self-worth, and economic motivation was 48.76 (SD = 14.56), 25.20 (SD = 9.03), and 24.77 (SD = 10.35), respoecilalu, Participants who committed to their current job had a higher work motivation score (both of three sub-scales) than those who not/unsure committed to the current job, the statistically significant difference with p < 0.001.

**Table 4:** Recent changes in work and work motivation of participants

Characteristics	Work retention						
	Not commit to current job/Unsure		Commit to current job		Total		p-value
	n	%	n	%	n	%	
**Commit to current job**							
Certain not					183	3.20	
Very little possibility					343	5.99	
Unclear					1,545	26.98	
Very much possibility					2,064	36.04	
Definitely yes					1,592	27.80	
	**Mean**	**SD**	**Mean**	**SD**	**Mean**	**SD**	**p-value**
**Recent changes in work** (range: 1–5)							
Daily work intensity	2.55	1.00	2.52	1.00	2.53	1.00	0.24
Level of work-related stress and fatigue	2.56	1.02	2.50	1.03	2.52	1.02	0.01
Health risks caused by work	2.48	1.02	2.36	1.04	2.41	1.03	< 0.001
The community’s stigma with the work I’m doing	2.17	1.04	1.89	1.01	1.99	1.03	< 0.001
Your ability to endure and cope with external work pressures	2.41	1.03	2.25	1.00	2.31	1.01	< 0.001
Process and professionalism of routine work	2.42	1.01	2.33	1.01	2.36	1.01	< 0.001
Complexity in coordination between colleagues, and between departments	2.38	1.02	2.24	1.02	2.29	1.02	< 0.001
New knowledge and skills for work	2.55	1.00	2.55	1.02	2.55	1.02	0.94
Ability to complete assigned tasks	2.46	1.01	2.36	1.05	2.39	1.04	< 0.001
Ability to ensure safe means of work	2.41	1.01	2.35	1.06	2.37	1.05	0.01
**Work motivation**							
Intrinsic motivation	42.31	15.36	52.41	12.71	48.76	14.56	< 0.001
Self-worth motivation	22.65	9.03	26.64	8.71	25.20	9.03	< 0.001
Economic motivation	22.34	10.14	26.15	10.21	24.77	10.35	< 0.001

Table 5 presents the Coefficient (Coef.), Odd ratio (OR), and 95%CI from Tobit and Ordered logistic regression analysis. The analysis highlighted the following significant associations: female participants were likely to have a higher intrinsic motivation score than male participants (Coef. = 1.08; 95%CI = 0.23; 1.93). Compared to the students, the nurse, and the technician tended to have a lower intrinsic motivation score, but have higher economic motivation score than the students (“Intrinsic” Coef. = 2.99; 95%CI = -5.00; -0.98; “Economic” Coef. = 2.40; 95%CI = 0.98; 3.82); meanwhile, doctor had a lower intrinsic motivation score (Coef. = -3.09; 95%CI = -5.32; -0.86). People who had the main income per month from 5 to 10 million VND (Coef. = 1.29; 95%CI = 0.10; 2.48) or more than 10 million (Coef. = 2.68; 95%CI = 0.94; 4.42) was likely to have a higher economic motivation score than those who had income less than 5 million VND. People who participated in COVID-19 pandemic prevention had a tendency to lower economic motivation, but have a higher intrinsic motivation than those who did not participate in it. Changes in daily work intensity, new knowledge, and skills for work were the positive factors of motivation that increased the intrinsic motivation score, by contrast, changes in the community’s stigma with the work, complexity in coordination between colleagues, and between departments had the opposite effect. Some factors affecting the commitment to the current job after adjustment are shown as follows: Married people tend to be more committed to their current job (OR = 1.91; 95%CI = 1.35; 2.72); Experience from 1 to 5 years or more (1–5 years: OR = 1.28; 95%CI = 1.04; 1.59; >5 years: OR = 1.56; 95%CI = 1.09; 2.24); Highly internally motivated people are more likely to commit to work (“Intrinsic motivation”: OR = 1.06, 95%CI = 1.06; 1.07).

**Table 5:** Factors associated with motivation among health professionals and medical students

Factors	Motivation						Commitment to current job	
	Intrinsic motivation		Self-worth motivation		Economic motivation		From 1 “Certainly not” to 5 “Definitely yes”	
	Coef.	95%CI	Coef.	95%CI	Coef.	95%CI	OR	95%CI
**SOCIO-ECONOMIC**								
**Gender** (Female vs. Male -Ref)	1.08**	0.23; 1.93	-0.44*	-0.91; 0.04				
**Marital status** (Married vs. Singled)							1.91***	1.35; 2.72
**Major** (vs. Student -Ref)								
Nurse, technician	-2.99***	-5.00; -0.98	0.12	-0.99; 1.23	2.40***	0.98; 3.82	1.06	0.75; 1.48
Doctor	-3.09***	-5.32; -0.86	0.15	-1.09; 1.40	1.31*	-0.19; 2.80	0.72*	0.51; 1.02
Other	0.46	-2.47; 3.38	-1.71**	-3.33; -0.09	1.96*	-0.17; 4.09	1.70**	1.02; 2.82
**Main income/month** (vs. < 5 million VND -Ref)								
5–10 million VND					1.29**	0.10; 2.48		
10 million VND or above					2.68***	0.94; 4.42		
**WORKPLACE INFORMATION**								
**Work experiences** (vs. Students -Ref)								
< 1 years	-1.71	-3.77; 0.36			1.91**	0.32; 3.51	1.12	0.78; 1.61
1–5 years	-1.07*	-2.29; 0.14			0.45	-0.46; 1.36	1.28**	1.04; 1.59
> 5 years	-0.16	-2.26; 1.94			-0.96	-2.40; 0.48	1.56**	1.09; 2.24
**Participate in COVID-19 pandemic prevention** (vs. Do not participate in -Ref)								
< 1 month	1.53***	0.38; 2.68	0.59*	-0.05; 1.22	-2.46***	-3.34; -1.58	0.85*	0.70; 1.03
1–3 months	1.86***	0.83; 2.89	0.87***	0.29; 1.45	-3.60***	-4.39; -2.80	0.81**	0.68; 0.97
> 3 months	1.41*	-0.14; 2.96	0.66	-0.20; 1.51	-4.67***	-5.82; -3.51	0.94	0.71; 1.22
**Number of on-call shifts per week** (vs. 0 -Ref)								
1 day	-1.03*	-2.23; 0.16					1.28**	1.05; 1.56
2 days	0.62	-0.91; 2.15					0.96	0.74; 1.24
> 2 days	0.22	-1.18; 1.63					1.04	0.82; 1.32
**Having part-time job** (Yes vs. No -Ref)							0.77**	0.63; 0.94
**The average workload increased** (vs. No changed - Ref)								
Increased < 20%							1.00	0.83; 1.21
Increased 20-<50%							1.08	0.84; 1.39
More than 50%							1.63***	1.17; 2.27
**RISK OF EXPOSURE TO COVID-19**								
**Get vaccinated against COVID-19** (vs. None)								
1 dose					1.14**	0.06; 2.22		
2 doses					1.04*	-0.05; 2.14		
**The current risk of exposure to COVID-19** (vs. None -Ref)								
Daily			-0.12	-0.90; 0.66				
Several times/weeks			-1.39***	-2.37; -0.42				
Seldom			-0.34	-0.95; 0.27				
Do not know			-0.13	-0.73; 0.46				
**Self-assess risk of COVID-19** (vs. None -Ref)								
Low risk	-0.33	-1.29; 0.64			-0.24	-0.98; 0.51	0.97	0.82; 1.14
Moderate risk	0.64	-0.59; 1.86			-1.20**	-2.14; -0.25	0.73***	0.59; 0.90
High risk	1.30	-0.29; 2.89			-1.11*	-2.33; 0.11	0.94	0.71; 1.25
Infected with COVID19/May be infected	-2.08*	-4.51; 0.36			0.81	-1.06; 2.69	0.80	0.53; 1.21
**Recent changes in work** (unit: score)								
Daily work intensity	0.59**	0.07; 1.10					1.10*	1.00; 1.21
Level of work-related stress and fatigue					0.30	-0.11; 0.71		
Health risks caused by work							0.90**	0.81; 1.00
The community’s stigma with the work I’m doing	-1.38***	-1.85; -0.90	0.59***	0.31; 0.87	0.30	-0.07; 0.68	0.84***	0.77; 0.92
Your ability to endure and cope with external work pressures			-0.30*	-0.64; 0.05			0.92	0.82; 1.02
Process and professionalism of routine work			0.41**	0.01; 0.80				
Complexity in coordination between colleagues, and between departments	-1.20***	-1.79; -0.61	0.36*	-0.01; 0.73	0.39	-0.09; 0.86		
New knowledge and skills for work	1.25***	0.69; 1.81	-0.29*	-0.62; 0.04	-0.37	-0.86; 0.12	1.11*	1.00; 1.24
Ability to complete assigned tasks					-0.44	-0.96; 0.09	0.89**	0.80; 0.99
Ability to ensure safe means of work					0.66***	0.17; 1.16		
**Change in job satisfaction** (vs. Decreased -Ref)								
Unchanged	-1.15**	-2.23; -0.06						
Increased	1.15*	-0.18; 2.49						
**Change in job motivation** (vs. Decreased -Ref)								
Unchanged	-2.33***	-3.45; -1.20	0.42	-0.09; 0.93	0.32	-0.39; 1.03	0.76***	0.65; 0.89
Increased	2.14**	0.98; 3.30	-0.07	-0.64; 0.49	-0.56	-1.34; 0.22	1.32***	1.11; 1.57
**Motivation** (Unit: score)								
Intrinsic motivation			0.33***	0.31; 0.35	0.13***	0.10; 0.16	1.06***	1.06; 1.07
Self-worth motivation	1.02***	0.96; 1.08			0.71***	0.66; 0.76	0.99	0.98; 1.00
Economic motivation	0.21***	0.16; 0.26	0.37***	0.35; 0.40				

*** p < 0.01, ** p < 0.05, * p < 0.1

## Discussion

This study is of importance as it hasb assessed the impact of COVID-19 on the career choices and intentions to transition to another career amongst Vietnamese healthcare workers. Only 17.2% of the respondents have had increased job satisfaction and 26.5% reported increased motivation to work. Most of the respondents (90%) have been vaccinated against COVID-19, and a good proportion (41.9%) reported that they did not perceive any risk of exposure to COVID-19. Whilst there were changes in the daily work intensity and the level of work-related stress, the respondents we sampled did not intend to make a job or career transition despite the challenges that they faced in their respective work environments during COVID-19. From the results, we managed to identify baseline and work-related characteristics associated with workers’ motivation to work. Demographic variables like gender, whether one was a student or an existing healthcare worker, and income related to work motivation. The community’s stigma was a negative factor that declined intrinsic motivation as well as decreased work retention.

One of the key findings was that most of the respondents did not report an increase in their job satisfaction or their motivation to work (as only 17.2% had increased job satisfaction and 26.5% increased motivation to work). Most of the respondents also reported there is an increase in the intensity of their work and corresponding stress. This finding is not unexpected, given that COVID-19 has had a tremendous impact on those working at the frontlines, and this has been reported by prior research [14]. It is widely known that the onset of COVID-19 not only resulted in healthcare workers having to spend long hours at work, but healthcare workers also have had to negotiate other challenges at their workplace. For example, they have had to constantly make adaptations to the changing nature of their work, as such adaptations are necessary when there is new information about the virus or its variants [14]. Healthcare workers have had to deal with these changes, but emotionally, they have had to also deal with the increased mortalities [14]. All these challenges would compromise the amount of quality time that they could devote to their families [14].

One of the other key findings that arose was that we found that females were likely to have a higher intrinsic work motivation score as compared to males. One of the reasons we postulate for there to be higher intrinsic work motivation among females as compared to male is how the Vietnamese government has provided financial support of US$45 for female medical workers and medical students who have participated in the COVID-19 pandemic [15]. Our findings that there is enhanced motivation in female healthcare workers appear to be somewhat incongruent with the existing findings in the research literature. In a recent scoping review undertaken by Morgan et al. (2022), female healthcare workers were found to be at a heightened risk for exposure and infection, and increased workloads, but with decreased leadership responsibilities [16]. It was also highlighted how they needed to deal with increased caregiving needs and were more susceptible to mental health issues, such as that depression, anxiety, and post-traumatic disorder [16]. Other research studies by Alon et al. (2020) and Wenham et al. (2020) have also argued that female healthcare workers are more susceptible to burnout, as they have to cope with the work-life balance [17, 18]. There is a need for future studies to explore qualitative factors and reasons as to why female healthcare workers have enhanced intrinsic motivation during the times of the pandemic, as our findings appear contrary to what has been reported in previous research.

The nature of the work environment, and whether workers have the right skills and knowledge also affect one’s motivation. It is pertinent to not consider these factors merely in isolation. Tran et al. (2022) [6, 13] have proposed a model that demonstrated how the interaction of different factors would result in an increased toll on healthcare workers. Such factors include individual predisposing factors (like health status, family attachment, and security), psychosocial outcomes of healthcare jobs, and the working environment. Hence, whilst individual factors were found to be associated with motivation level, we need to also consider the inter-relationship between these factors. Understanding the inter-relations between these factors could help guide policy implementation. We concur with the recommendations that Tran et al. (2022) [6, 13] have articulated, such as better optimization of operational needs and coordination amongst services, building capacity, and perhaps also ensuring that workers have adequate and updated knowledge.

Lastly, and most importantly, our study revealed that there was an impact of stigma on healthcare workers’ motivation to work. This finding is congruent with a recent study undertaken by Do Duy et al. (2020), which sampled a total of 61 participants, of which the majority were nurses, and reported that the participants were concerned about their negative self-image and public attitudes [19]. It remains important for governmental organizations in recognizing the need to promote appreciation for healthcare workers so that they feel appreciated by the public.

There are several strengths of this current research. The main strength of this study is that it helped to bridge the gap in the current research literature, by exploring the impact of COVID-19 on career choices and transitions amongst healthcare workers. This study also managed to identify factors, such as demographic variables and others related to the nature of work/environment that moderate one’s motivation to work. We manage to recruit a large sample size through snowball sampling, and we managed to tap on online mechanisms for recruitment during the pandemic. Despite these strengths, there are several limitations. Participants were recruited using snowball sampling, using a web-based/online mechanism, and this might affect the overall representativeness of the sample as well as potentially introduce bias into the sample. We managed to recruit participants over a short period, and only during a specific period/wave of infection during the COVID-19 pandemic. As the pandemic progresses, it might have a further impact on participants, which might affect the results we present. We were limited to a cross-sectional study, and hence, we are not able to elucidate any causal relationships concerning the identified factors.

## Conclusions

Our study is instrumental in examining the impact of COVID-19 on career choices among Vietnamese healthcare workers. Whilst it is expected that the onset of the pandemic increased demands at work, most healthcare workers are inclined to remain at their workplaces. Several demographic and work-related variables affect one’s motivation.

## Electronic supplementary material

Below is the link to the electronic supplementary material.

Supplementary Material 1

## Data Availability

All data generated or analyzed during this study is available from the corresponding author on request.
