# Supplementary material for: Impacts of COVID-19 on career choices in health professionals and medical students
Source: BMC Med Educ. 2023 May 26;23:387. doi: 10.1186/s12909-023-04328-8 (PMC10215048; doi:10.1186/s12909-023-04328-8)
Supplement: Supplementary file 1 — Supplementary Material 1 [file 12909_2023_4328_MOESM1_ESM.docx]

**APPENDIX**

**Appendix 1. Work Motivation Scale**

**Please tell us your opinion about the comments below that relate to your work motivation with your current job during the COVID-19 pandemic:**

1) Because I enjoy doing what I do at work every day.

2) Because I enjoy my work tasks.

3) Because the work that I do is very interesting.

4) Because being a health worker is a fundamental part of who I am

5) Because my work is extremely important for my patients

6) Because I want to make a difference in people’s life

7) To feel good about me

8) Because my reputation depends on my work.

9) Because of the appreciation I receive from my patients and the community

10) Do not let my team down.

11) Because my supervisor recognizes and appreciates me

12) Because of the benefits that come with my job

13) To be able to provide for my family

14) Because of the financial security my job provides me with

15) To earn money
